# The clinical fellows project: emergence of the FY3 year?

**DOI:** 10.1192/bjb.2020.16

**Published:** 2020-04

**Authors:** Anirban Raha, Robert Heminway

**Affiliations:** CT2, Sheffield Health and Social Care NHS Foundation Trust, email: anirban.raha@shsc.nhs.uk; CT1, Sheffield Health and Social Care NHS Foundation Trust, email: robert.heminway@shsc.nhs.uk

Since August 2017, Sheffield Health and Social Care have begun providing a new non-training post in psychiatry. This was done with multiple aims, including attempting to increase the uptake of core psychiatry training jobs in South Yorkshire by giving a positive experience of working in a psychiatric setting and improving local clinical care with an increase in the number of doctors working at SHO/CT1 level. The non-training posts were described as clinical fellowships and were open to doctors who had not yet undertaken higher training in psychiatry.

We, Drs Raha and Heminway, were among the first people to take up these clinical fellowship posts and were therefore in a prime position to review how the project is being received and some of its outcomes. Since August 2017, 15 clinical fellows have been recruited, largely in the ‘FY3 gap’ but with some coming from general practitioner (GP) training. Dr Raha entered after having been a GP trainee and Dr Heminway from a post-foundation training break.

The clinical fellowship consists of a non-training junior doctor post, working at FY2/CT1-equivalent level in psychiatry. A number of rotational placements were created, including those based in general adult, older adult and addiction psychiatry, across both community and in-patient settings. A key benefit that was felt by the clinical fellows was that there were additional training opportunities given – a split of 80% clinical to 20% non-clinical time allowance, access to weekly continuing professional development (CPD) sessions, weekly supervision with a consultant psychiatrist and attending the core psychiatry training course for MRCPsych examinations. Clinical fellows have also since set up their own peer group to improve CPD and training opportunities for future clinical fellows.

The experience has been invaluable, not just for ourselves but also for other clinical fellows involved in the project; this has been borne out by the increasing uptake of core psychiatry training posts by the clinical fellows. Of the 2017–2018 cohort, half entered core psychiatry training, one entered GP training and one left owing to long-term sickness. Half the 2018–2019 cohort were also considering entering psychiatric training at the end of the clinical fellowship posting.

The culmination of this experience was the opportunity to present this project at the Choose Psychiatry meeting of the Royal College of Psychiatrists earlier this year. It was well received, owing to the fact that it was both improving access to psychiatry and improving junior doctors’ opinion of psychiatric training and work (including those leaving the post to take up GP jobs). It was also highlighted that many junior doctors are no longer entering core training directly after foundation training. This clinical fellowship has therefore been an excellent way to accommodate this trend and provide a springboard for increasing access to and awareness of psychiatric training.

